# A Comprehensive Review of Niosomes: Composition, Structure, Formation, Characterization, and Applications in Bioactive Molecule Delivery Systems

**DOI:** 10.3390/molecules30173467

**Published:** 2025-08-23

**Authors:** Alfredo Amaury Bautista-Solano, Gloria Dávila-Ortiz, María de Jesús Perea-Flores, Alma Leticia Martínez-Ayala

**Affiliations:** 1 Departamento de Ingeniería Bioquímica, Escuela Nacional de Ciencias Biológicas, Instituto Politécnico Nacional (IPN), Av. Wilfrido Massieu Esq. Miguel Stampa s/n, Zacatenco, Alcaldía Gustavo A. Madero, Ciudad de Mexico 07728, Mexico; abautistas2103@alumno.ipn.mx (A.A.B.-S.); gdavilao@yahoo.com (G.D.-O.); 2 Centro de Nanociencias y Micro y Nanotecnologías, Instituto Politécnico Nacional (IPN), Unidad Profesional Adolfo López Mateos, Av. Luis Enrique Erro s/n, Zacatenco, Alcaldía Gustavo A. Madero, Ciudad de Mexico 07738, Mexico; 3 Departamento de Biotecnología, Centro de Desarrollo de Productos Bióticos, Instituto Politécnico Nacional (IPN), Carretera Yautepec-Jojutla s/n-km 85, San Isidro 62739, Morelos, Mexico; alayala@ipn.mx

**Keywords:** nanotransporters, niosomes, niosome composition, niosome formation, drug delivery

## Abstract

Niosomes are nanocarriers with a bilayer structure, consisting of a polar region and a non-polar region. This unique structure allows them to encapsulate compounds with varying polarities, addressing solubility challenges in the transport and delivery of bioactive molecules. The formation of niosomes involves key structural, geometric, and thermodynamic factors influenced by the choice of surfactants and preparation methods. These factors, including the critical packing factor and the hydrophilic–lipophilic balance (HLB), play a crucial role in determining the properties of the final niosomes. Additionally, the use of Tandford’s equations allows for the calculation of geometric parameters. These factors determine the structural integrity and functional properties of niosomes, making it possible to design functional niosomes with characteristics tailored for specific applications. This ability to design niosomes with desired properties is especially valuable in biomedical fields, where precise control over drug delivery and targeting is essential. This review highlights the importance of niosome formulation and presents examples of niosomes that have been functionalized for specific applications, including anticancer treatments, immunological treatments, and their action in the central nervous system.

## 1. Overview

There are various molecules with biological activity and relevant applications such as antioxidants, anticancer agents, antibacterial agents, and antiviral agents. However, many of these molecules show low absorption, physicochemical instability, and uncontrolled release [1,2,3]. This means that bioactive compounds degrade or lose their effectiveness before reaching the specific site where their therapeutic function should be exerted. For this reason, developing effective strategies for the transport and proper administration of these molecules is essential in the pharmaceutical field [4].

Molecular transport is defined as the ability of a carrier system to accurately guide a bioactive compound to a target location without significant interference or loss during the process [5]. An effective transport system must not only be capable of preserving the stability of the encapsulated molecule but also ensure controlled and specific release, minimizing leaching or loss of the active compound before its destination is reached. In addition, it becomes essential that these delivery vehicles are biocompatible and do not generate adverse effects in the body [6,7].

Molecule transport systems, mainly divided into inorganic and organic, are classified according to their structure and composition [8]. Niosomes fall within organic lipid systems. Niosomes are lamellar systems composed of a bilayer with amphiphilic characteristics, i.e., they include both hydrophilic and lipophilic domains. This property allows them to encapsulate and transport molecules with different polarities, increasing their range of applications, making them a versatile and promising option [9,10].

Niosomes are mainly composed of nonionic surfactants and cholesterol, which interact to form a stable bilayer. Despite their robustness, niosome formation requires a hydration medium with suitable conditions and, in some cases, charge inducers to prevent fusion between particles and ensure better system stability [11]. Niosomes can vary in size, number of layers, and functionality, depending on factors such as pH, the physicochemical properties of the surfactant used, and the synthesis method employed [12].

Niosome design is an important issue, as these variables determine the final system properties, such as stability, encapsulation efficiency, controlled release, size, and polydispersity, which are key aspects for specific applications [13].

The applications of niosomes are wide-ranging, from drug delivery and gene therapy to the release of cosmetic agents and antioxidants. Their ability to encapsulate sensitive compounds, protect them from premature degradation, and efficiently deliver them to the target site makes them essential tools for personalized medicine and the development of advanced therapies [14,15]. However, niosome design must be adjusted according to the characteristics of the molecule to be transported and the specific needs of the application. Niosomes represent an innovative solution to overcome bioavailability limitations and bioactive molecule stability. Their structural and functional versatility, combined with an optimized design, positions them as one of the most promising strategies in the development of controlled release systems, with applications in scientific and technological fields [4,16,17].

## 2. Niosome Structure

**Figure 1:**
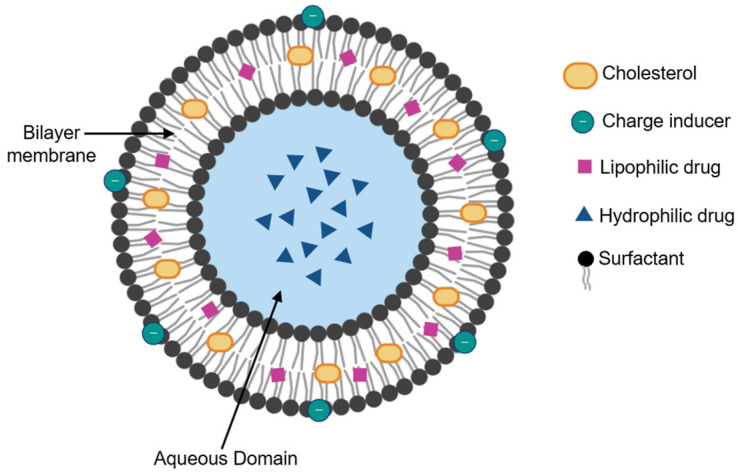
Bilayer structure of niosomes. This image was created using Biorender (https://biorender.com/pyili16, accessed on 5 June 2025).

Niosomes are generally composed of cholesterol, surfactants, charge inducers, and a hydrating medium, which, when mixed in the right proportion, provide stability to the vesicle, forming a bilayer structure. This bilayer gives niosomes a particular geometry with two areas: a central one giving an aqueous domain and another lipid domain. Niosomes can encapsulate both hydrophilic and hydrophobic drugs. Hydrophilic drugs are encapsulated in the central aqueous domain, while hydrophobic species are encapsulated by partitioning, as shown in Figure 1 [2,4].

## 3. Niosome Components

Components to be encapsulated in niosomes are important because their structural and functional characteristics depend on them. Niosomes are formed from four fundamental components (surfactants, stabilizers, charge-inducing molecules, and the hydration medium [1]).

### 3.1. Surfactants

Surfactants, also known as surface-active agents, are structures capable of modifying the liquid surface tension and the interfacial tension between two liquids [9].

Surfactants are generally organic molecules containing charged and uncharged groups, giving them an amphiphilic nature. Groups with a net electric charge are found in polar zones known as “polar heads” because there are groups that have a noticeable dipole moment, such as sulfanates (SO_3_^−^), phosphates (PO_4_^−^), carboxylates (COO^−^), primary ammonium (-NH_3_^+^) and quaternary ammonium (R-NR_3_^+^X^−^) groups, as well as other polar groups such as carbonyl derivatives, siloxanes, polyether groups with ethoxylated sequences, and others. On the other hand, the nonpolar area is the area with no net charge, i.e., formed by atoms not generating a dipole moment, generally comprising linear or branched carbon chains. Due to these characteristics, a surfactant is amphiphilic by having the ability to have a certain affinity for polar solvents (water, glycols, amines, alcohols) and non-polar solvents (aromatic solvents, alkanes, and others) [9].

Surfactants in low concentrations exist in monomer form when dissolved in polar solvents such as water. As the concentration increases and reaches an optimal value, these monomers group together in such a way that hydrophobic parts (tails) are oriented towards the center of the micelle, and hydrophilic parts are positioned towards the solvent, helping to reduce the surface tension between phases [5].

Types of surfactants:

Different types of surfactants are classified according to their chemical structure and the type of charge on their hydrophilic part.

#### 3.1.1. Ionic Surfactants

Hydrophilic surfactants are highly polar in nature and soluble in water and polar solvents; lipophilic surfactants contain long carbon chains, resulting in a high degree of lipophilicity. Ionic surfactants are surfactants containing electrically charged atoms or molecules, i.e., ions resulting from electron deficiency or deprotonation of functional groups [18]. This type of surfactant is further subclassified according to the type of ions generated. Cationic surfactants are those containing a positively charged functional group in their polar part, such as quaternary ammonium salts. These surfactants maintain their positive charge throughout the pH scale. Their main function is based on their ability to adsorb onto surfaces and interact with negatively charged compounds, being useful as emulsifying agents and surface modifiers, also having applications in improving the solubility of certain substances. On the other hand, anionic surfactants contain negatively charged groups such as sulfates (-OSO_3_^−^), sulfonates (-SO_3_^−^), phosphates (-PO_3_^−^), and carboxylates (-COO^−^). The latter are the most common groups, comprising carboxylate salts in the form of sodium stearates and fluorides like fluoroalkyls such as C8F17, which enhance the stability of vesicles by modifying the surface charge of niosomes. A third class are zwitterionic surfactants, having a hydrophilic part with two groups, one positively charged and the other negatively charged, meaning that they have cationic and anionic centers in the same molecule, keeping it neutral [9].

#### 3.1.2. Nonionic Surfactants

Nonionic surfactants do not contain dissociable functional groups, so ions are not produced. These types of surfactants contain hydrophilic groups covalently bonded to hydrophobic parent structures. Hydrophilic groups generally contain oxygen, causing intermolecular interactions such as hydrogen bonds, increasing their solubility in polar protic solvents such as water. It is important to mention that these are less sensitive to water hardness and generally have an ideal hydrophilic–lipophilic balance for micellar structure formation [18].

In addition to the abovementioned surfactant types, there are others with particular characteristics, such as symmetrical surfactants. These types of surfactants contain hydrophilic groups located at the ends and connected by a spacer group, i.e., functional groups that can form covalent bonds between the surfactants [11].

### 3.2. Cholesterol

Another component of niosomes is cholesterol. Cholesterol is a cyclopentanoperhydrophenanthrene, basically considered a sterane. Its structure is made up of four fused carbocycles named A, B, C, and D. It has substitutions at the C-10 and C-13 positions corresponding to methyl groups, contains one secondary alcohol at the C-3 position, is unsaturated between the C-5 and C-6 carbons, and has an aliphatic chain of eight carbons at the C-17 position [12].

Cholesterol is related to different characteristics of niosomes. It influences physical properties because it affects membrane cohesion and mechanical resistance, reduces membrane flexibility, regulates drug penetration, improves encapsulation and stability, and increases the phase transition temperature. The surfactant–cholesterol ratio can affect niosome structure, producing higher viscosity niosomes [1,13]. A linear increase in carboxyfluorescein encapsulation was reported, which increased its encapsulation efficiency as the cholesterol concentration increased [1,13].

### 3.3. Charge-Inducing Molecules

Charge-inducing molecules are those with functional groups providing a positive or negative charge. These molecules stabilize membranes and prevent aggregation by electrostatic repulsion, preventing fusion between niosomes [15,16]. The effects of charge inducers are reflected in zeta potential, which can be taken as a parameter of niosome stability. Niosomes with a zeta potential greater than +30 mV or less than −30 mV indicate highly stable niosomes. Values between 5–15 mV represent the flocculation limitation region, i.e., niosome agglutination, and values between 5 and 3 mV indicate maximum flocculation [19].

There are different charge inducers, such as dicyclopentyl phosphate and phosphatidic acid, which are negatively charged ionic compounds. Positive charge inducers can be stearyl amine, sodium stearoyl lactate, and stearylpyridine chloride in their cationic form. These inducers are added to the formulation in an amount of approximately 2.5% to 5% because higher amounts than those mentioned can prevent niosome formation [16].

Another reported effect of charge inducers is that they are related to encapsulation efficiency. An example is the addition of sodium stearoyl lactate (SSL), which increases niosome encapsulation efficiency due to electrostatic interaction between the negatively charged SSL group and the positively charged drug residue [1].

Charge-inducing molecules are also related to the size that may be reached by a niosome. Positively charged drugs neutralize the anionic molecule charge; thus, niosomes will be smaller than cationic vesicles because of repulsion between drug anionic charges and the charge inducer. This charge induction also improves skin permeation in niosomes intended for use as ointments [20].

### 3.4. Hydration Medium

The hydration medium is another niosome additive required for the hydration phase. Buffers are generally used, considering that the buffer pH depends on the encapsulated drug’s solubility. An example of this is niosomes synthesized for ketoconazole encapsulation, where a phosphate buffer (PBS) with a pH of 5.5 was used. Understanding the physicochemical properties in the formulation of niosomes is essential to obtain nanovesicles with the ability to encapsulate and transport the desired drug or biomolecule [4].

## 4. Niosome Formation

Amphiphilic molecules such as surfactants have the ability to associate in various structures that can transform from one to another when solution conditions are modified.

These factors can include ionic strength, type of solution ions, pH, and temperature. In addition to these factors, system thermodynamics and intra- and intergroup forces are involved, which determine the equilibrium structures that can be formed. Amphiphilic structures can be created, which may exist in a non-equilibrium state, meaning a state that has not reached its lowest energy form and thus is not in thermodynamic equilibrium, through controlled assembly using physical and chemical processes. A non-equilibrium structure is one that does not follow the Gibbs’ phase rule, describing the number of degrees of freedom in a closed system at equilibrium, having several separate phases and a number of chemical components in the system [21].

Micelles, bilayers, and three-dimensional networks are structures formed due to forces governing their self-assembly. These derive from the hydrophobic attraction at a hydrocarbon–water interface and the ionic or steric hydrophilic repulsion induced by the head groups. These two types of interactions compete as two opposite proportional forces in the interfacial region. The attractive interaction arises mainly from hydrophobic or interfacial tension, represented by a positive interfacial free energy per unit area, which is a characteristic of the interface that is reduced in the presence of a hydrophilic group. On the other hand, repulsive forces are very complex and therefore difficult to formulate experimentally. However, different contributions such as steric repulsion, hydration force, and double-layer electrostatic force may be considered if the molecule’s head group is charged. With these factors, energy expansion can be expected to be inversely proportional to the surface area occupied by each head group [22].

## 5. Factors Involved in the Niosome Formation

Niosome formation depends on several physicochemical factors, the reagents being used, and the synthesis methods. This results in niosomes with different geometric characteristics, sizes, stability, and functions.

### 5.1. Thermodynamics of Niosomes

Niosomes are self-assembling structures stabilized by various intermolecular interactions such as Van der Waals forces, dipole–dipole interactions, hydrogen bonding, and electrostatic forces. The magnitude and nature of these interactions depend both on the chemical structure of the surfactant used and on the supermolecular assembly geometry. From a thermodynamic point of view, the self-assembly process, as well as the principle of micellization, is governed by Gibbs free energy, which can be described by the Gibbs–Helmontz equation [23].

$${∆G}_{m}={∆H}_{m}-T{∆S}_{m}$$

where

$∆H_{m}$ = molar enthalpy change (J/mol);

T = temperature in Kelvin degrees (K);

$∆S_{m}$ = molar entropy chang (J/mol·K).

The above equation expresses the molar Gibbs free energy changes (∆G_m_) of the system, (∆H_m_) represents the molar enthalpy change associated with the assembly, T is the absolute temperature, and ∆S_m_ is the molar entropy change. This is because for self-assembly to occur spontaneously, ∆G_m_ must be negative. State functions such as enthalpy and entropy result from micellization at a given temperature. Micellization, being a process of molecular ordering and packing, produces an unfavorable change in entropy caused by steric or electrostatic repulsion between the polar parts of the molecule.

In the case of nonionic surfactants, thermodynamic equilibrium is closely related to critical micelle concentration (CMC). Above the CMC, additional surfactant molecules are incorporated into existing micelles, leading to an increase in the size of the aggregate structures. Micellization involves a molecular reorganization that may represent a local decrease in entropy, due to polar region packing or steric or electrostatic repulsions. However, the overall process usually includes net entropic gain, mainly due to the release of water molecules structured around hydrophobic chains [24].

Niosome formation is a non-spontaneous process, since thermodynamically stable vesicles will only be formed in the presence of specific mixtures of surfactants and charge-inducing agents. Niosome formation is an energy-demanding process, achieved through different means such as stirring, heating, and pressure. Different thermodynamic and physicochemical parameters must be considered, such as hydrophilic–lipophilic balance, molecular geometry, surface energy, mechanical energy, chemical energy, and potential energy [23].

The association of monomers and nonionic surfactants during the dehydration phase within the synthesis process indicates high interfacial tension between water and the hydrocarbon portion. This high interfacial energy causes the association of different groups. Steric effect and ionic repulsion indicate that the polar part of the molecule contacts with water, and this interaction results in supramolecular assembly. An adequate concentration of surfactants must be considered since, at low concentrations, asymmetric micelles are formed, and concentrations greater than 70% will result in lamellar liquid–crystalline interactions. To define the thermodynamic energy associated with micelle formation (E), it is interpreted with surface energy (Ey), mechanical energy (EΔp) due to curvature, and variation in chemical potential excess (EΔμ’). Thermodynamically, the system expresses negative entropy (−ΔS) and a reduction in free energy (−E°), i.e., the system shows greater internal organization and a spontaneous tendency towards a more stable and energetically favorable state. This is only achieved through a favorable enthalpy contribution (−ΔH) arising from Van der Waals forces, hydrophobic forces, hydrogen bridge interaction, and the shielding of electrostatic interactions. The formation of small niosomes requires a high energy input, creating a thermodynamically unfavorable packing state. The fusion of these vesicles is a system by which the system dissipates excess surface energy originating from the packaging. The synthesized niosomes contain complex mixtures, thus making it important that their behavior in the presence of organic agents and surfactants is considered [25].

### 5.2. Geometry of Niosomes

Like all amphiphilic molecules, niosomes have more favorable structures; however, the molecular geometry of the surfactant can impose packaging restrictions. Packaging properties are determined by the optimal geometric arrangement of amphiphilic structures. This is determined by the critical packaging parameter (CPP), which is a dimensionless number that provides information about interactions occurring between apolar and polar regions. These regions, in turn, determine the limiting shapes that molecules can adopt and their assembly. Therefore, CPP provides information about the type of structure that will be formed according to established ranges [24].

This value depends on its optimal polar area (α_0_), non-polar volume (ν), and the length of its hydrocarbon chain. The CPP can be calculated using various chemical software programs (Vega ZZ (version 3.2.4.28), RDkit (version 2025.03.3), Gaussian (version 16), ORCA (version 6.1.0)). Another way to do this is by using Tanford equations to estimate the properties of alkyl chains in amphiphilic molecules. These equations allow the calculation of the non-polar chain length, polar area, and non-polar volume based on the number of carbons in the alkyl chain [22].

### 5.3. Non-Polar Length

The non-polar length (l_c_), also called the critical chain length of the hydrocarbon tail, is defined as the total distance in Angstroms of the hydrophobic part of the molecule. The critical length is a semi-empirical parameter, representing a limit on how far the chains can extend, allowing shorter but not larger extensions, because they are entropically costly and cannot be considered fluid. This length is calculated by considering the number of carbons in the chain [26].

$$l_{c}=(0.154+\left(0.1265\cdot n\right))$$

where

l_c_ is the non-polar chain length in angstroms;

n is the number of carbon atoms.

### 5.4. Polar Area

The polar area depends on the degree of hydration of the polar head, and the degree of ionization further influences charged amphiphilic structures. This parameter is related to the niosome structure curvature; a large polar area, compared to the length of the nonpolar chain, favors the formation of small, spherical micelles, while smaller areas may favor flatter structures such as bilayers [21].

Tandford proposes ranges of polar areas depending on the type of surfactant used, ranging from 40–70 Å^2^. For example, spans (sorbitan esters), Tweens (polysorbates), and pluronics are nonionic surfactants widely used in emulsion formulations.

### 5.5. Nonpolar Group Volume

The non-polar group volume, also called micelle core volume, indicates the physical space lacking a dipole moment in that molecule portion, excluding polar groups such as hydrophilic heads, which will depend on the number of tails, the presence of unsaturated bonds, and branching.

The non-polar volume is calculated using the following equation.

$$V=27.4\cdot n{Å}^{3}$$

where

n is the number of carbon atoms;

27.4 Å^3^ is the approximate volume occupied by each methylene unit in the chain (-CH_2_).

**Figure 2:**
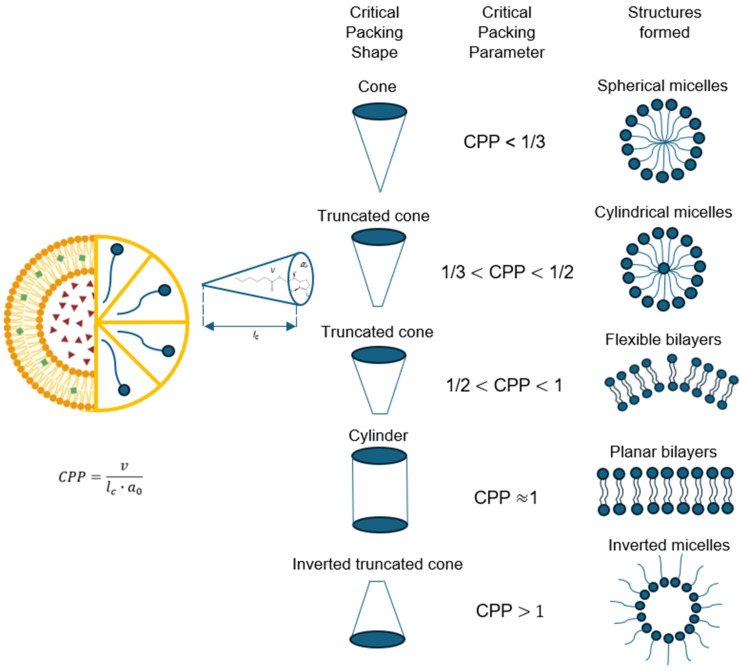
Mean packing shapes and the structures they form.

A wide variety of structures may be formed under the same critical packing parameter, as illustrated in Figure 2. However, entropy favors the structure with the smallest number of aggregated molecules because it will have less free energy. In other words, larger structures are entropically disadvantaged and small structures increase the surface area above an optimal value, making them energetically disadvantaged. Depending on this value, amphiphilic structures can be organized from spherical and cylindrical micelles to inverted structures according to the CPP value ranges [26].

The formation of spherical micelles is reserved for structures where the micelle core radius does not exceed a value between 80–90% of the critical chain length. This condition is only met when the CPP ≤ 1/3. Niosomes that adopt spherical structures are those that contain a large polar area. Another case is when amphiphilic structures have a small polar head compared to the critical chain length, which fits the parameter 1/3 ≤ CPP ≤ 1/2, resulting in cylindrical micelles in different cylindrical shapes, either worm-like or rod-like. Cylindrical worm-shaped micelles cause viscosity increases in the solution, while cylindrical rod-shaped micelles change with the ionic strength or pH of the aqueous phase. Double-tailed surfactants can adopt a cone shape when they have 1/2 ≤ CPP ≤ 1 values and self-assemble into spherical bilaminar vesicles. Inverted micelles are a type of geometry obtained by lamellar and flat assemblies, where CPP > 1, which generally adopt highly hydrophilic surfactants. The structures described may have greater aggregation as the concentration of the surfactant increases, leading to the formation of different mesophases, cubic, bicontinuous cubic, hexagonal, and lamellar phases [27].

Each of these structures is the one with the lowest energy, i.e., its most stable configuration. In concentrated systems, interactions between aggregates cause transitions to ordered structures called mesophases. These structures are driven by short-range repulsive forces between the aggregates. As the concentration of amphiphiles increases, the water content and the optimal area of the molecules decrease, driving the structures towards higher values and making them more ordered. One factor that modifies the shape is the concentration of surfactant. At high surfactant concentrations, spherical micelles tend to transform into hexagonal cylinders, cylindrical micelles transform into interconnected cubic structures, and extended bilayers transform into inverted structures. These changes are called first-order phase transitions that occur between single-phase regions separated by two-phase regions [25].

In view of the above, it is vitally important to select a suitable surfactant in niosome formulation. This is determined by its hydrophilic–lipophilic balance (HLB) value and the Critical Packing Parameter (CPP), in addition to the phase transition temperature. HLB determines niosome encapsulation efficiency; the HLB value is directly proportional to the length of the molecule’s hydrocarbon chain, which is the critical length that will be assumed as fluid or deformable. A high HLB value indicates a long alkyl group chain and therefore large niosomes. Similarly, the transition temperature is a critical point in encapsulation efficiency, as it involves the phase transition from gel to liquid crystal [22].

### 5.6. Hydration Temperature

The hydration temperature influences the formation of niosomes, as well as their shape and size. The temperature at which niosomes should be during the hydration stage must be above the gel-to-liquid phase transition. This temperature determines the formation of niosomes. For example, temperatures between 45–48 °C form large spherical niosomes, while temperatures above 55 °C form smaller spherical niosomes. There is an ideal temperature range of 70–75 °C to produce small, uniform niosomes [28].

### 5.7. Effect of pH

Micelles are formed in aqueous solution through the self-assembly of amphiphilic molecules. Unlike solid, rigid particles such as viruses, globular proteins, and DNA, niosomes are soft and flexible, with characteristics similar to a fluid. This is because the forces that hold amphiphilic molecules together are not covalent or ionic bonds but arise from Van der Waals, hydrophobic, hydrogen bond, and electrostatic interactions. Because of this, if the solution conditions, such as the pH, are modified, the interactions, size, and shape of the aggregates will be affected. This is because pH affects ionization of functional groups. In acidic conditions, micelles can disintegrate due to protonation of functional groups. On the other hand, in basic solutions, deprotonation can cause greater repulsion between molecules, affecting their ability to form [29].

pH also influences the critical micelle concentration (CMC), referring to the minimum surfactant concentration needed for micelle formation. This depends on the pH value; as the pH changes, the molecule’s charge changes, affecting its solubility and stability. An increase in pH reduces CMC for certain surfactants [30].

Niosomes contain nonionic surfactants with (-OH^−^ and -O^−^) groups in their structure, as well as aliphatic or aromatic chains that allow them to interact with water without ionizing. However, at extreme pH values, the stability and size of niosomes can be affected due to electrostatic interactions that may arise. A balanced pH promotes adequate interaction between components [29]. The pH of the hydration medium is related to encapsulation efficiency because it interacts with the wall material and the molecule to be encapsulated. For example, flurbiprofen is efficiently encapsulated at a pH of 8 to 5.5. This is explained by the molecular structure of flurbiprofen, which contains acidic hydrogens [1]. Another example is niosomes synthesized using Tween 20 and glycine for the encapsulation of ibuprofen in acidic environments [31].

## 6. Preparation Methods

The methods used to prepare niosomes affect their shape and size, and these methods must be considered to obtain niosomes with the desired characteristics.

### 6.1. Solvent Injection

This method of niosome synthesis involves the use of organic solvents such as ethanol or ethoxyethanol, also known as ethyl ether. Like other methods, the use of cholesterol and a surfactant is involved. The mixture with the selected organic solvent is slowly added to a preheated aqueous solution containing the drug. A phase transition is carried out by maintaining this solution at a constant temperature above 60 °C. As the solvent evaporates, vesicles of different sizes ranging from 50–1000 μm are formed, and it can be said that this method is focused on obtaining multilamellar niosomes transporting hydrophilic drugs [31].

### 6.2. Bubble Method

All niosome components are used in their design; however, in this method, a phosphate buffer is preferably used as the hydration medium, and this buffer’s pH is determined according to the drug to be encapsulated. This mixture is added to a three-necked flask, maintaining the temperature in a water bath. Nitrogen is passed through one of the necks, a water-cooled reflux is attached to another neck, and, finally, a thermometer is placed in the third neck to monitor the temperature. Components are dispersed at 70 °C and homogenized for 15 min. The produced niosomes are large unilamellar niosomes [32].

### 6.3. Reverse Evaporation Phase Method

This method is generally used to encapsulate hydrophilic drugs. The mixture of surfactant, charge inducers, stabilizer, and organic solvent is added to an aqueous solution containing the drug to be encapsulated. The resulting system is homogenized, and the organic phase is removed by negative pressure, yielding niosomes of different sizes [33].

### 6.4. Membrane pH Gradient

Molar ratios between cholesterol and surfactant must be taken in equal measures for this method by dissolving them in organic solvents with a polarity like chloroform. The organic solvent is removed by reduced pressure, forming a thin lipid layer on the flask’s inner surface. The lipid layer is hydrated using a citric acid solution or any other acid with a similar pKa value. The resulting niosomes are subjected to freezing and thawing processes. A drug aqueous solution is then added and mixed with the aid of a vortex. Finally, the pH is adjusted using a phosphate solution to obtain large unilamellar niosomes. High encapsulation efficiencies are achieved with this type of niosome [34].

### 6.5. Heating Method

The heating method differs from the others in that the hydration stage is carried out individually for both cholesterol and the nonionic surfactant using a buffer solution. The solution is heated for 1 h at 120 °C to dissolve cholesterol. Once the cholesterol has been solubilized, the surfactant and other additives are added under continuous stirring, producing the niosomes, which stand at room temperature for 30 min, resulting in small unilamellar niosomes [35].

### 6.6. Freezing and Thawing Method

This method aims to obtain unilamellar vesicles through post-treatment using various methods, which are an extension of the pH gradient and thin film hydration methods, among others. Freezing cycles are carried out using liquid nitrogen for 1 min and thawing in a water bath at a temperature of 60 °C to reduce the size of the niosomes. However, freezing and thawing cycles affect niosome encapsulation efficiency [36].

### 6.7. Microfluidic Hydrodynamic Approach

This method uses miscible solvents through a microfluidic hydrodynamic approach by diffusive mixing. Solvents are rapidly mixed in microchannels in a controlled manner. Optimal values are achieved in polydispersity and size with this method for niosomes. The nonionic surfactant chemical nature and the microchannel device material to be used are mainly considered for this end, which causes an increase in the sample processing time and therefore affects the size of the niosome [37].

### 6.8. Dehydration–Rehydration Method

This method combines three niosome synthesis techniques. First, a lipid film is formed, which is then frozen in liquid nitrogen and lyophilized. Powdered niosomes are hydrated with a saline solution at a pH of 7.4 at 60 °C, thus producing unilamellar niosomes used in eye treatments. This method also increases encapsulation efficiency, being one of the most efficient methods in this parameter [38].

### 6.9. Thin Film Hydration Method

Also known as the stirring method. This is the simplest way to obtain niosomes. An organic solvent with polarity, like chloroform, is used for this method, in which surfactants, charge inducers, stabilizers, and the drug to be used are mixed in specific molar ratios in a round-bottom flask. The solvent is then evaporated in a rotary evaporator to form a lipid film, which is hydrated using water or phosphate buffer. This hydration forms multilamellar vesicles containing the drug or biomolecule [39,40].

## 7. Nanoparticle Dispersion and Reduction

The size, homogeneity, and dispersion of niosomes can be controlled using different techniques with the aim of decreasing their size and presenting unique physicochemical and biological characteristics. A post-treatment of synthesized niosomes is required. The most common methods for reducing the size of niosomes are extrusion and ultrasound [33,41].

### 7.1. Extrusion

The extrusion method consists of a deformation process aimed at molding a material at high or low temperatures by forcing it through a die (mold that defines the shape and dimensions of the final product) with the geometry and dimensions of the desired product. Three basic steps are involved: gathering the raw material in a solid and molten state, continuously melting the raw material, and homogenizing the raw material thermally and physically. Specifically in relation to niosomes, nanoextrusion methods have been used, consisting of passing these amphiphilic structures through membranes to obtain large or small unilamellar vesicles in the order of 10–250 nm [42].

### 7.2. Ultrasound

The application of ultrasound produces a cavitation effect that disperses and reduces materials to break up agglomerates using a sonotrode in a fluid. The sonotrode generates a vapor film through small bubbles that expand and contract until they collapse, generating cavitation due to an increase in pressure, producing a shock wave that breaks up the agglomerates. Ultrasound is considered an energy source that is used in various research fields, such as medicine and biotechnology, among others, with applications in homogenization, emulsification, extraction, crystallization, and degassing. Ultrasound has advantages in multiple processes, especially in terms of speed, selectivity, reproducibility, and energy savings. Thus, it is considered a source of green technology that complies with the 12 principles of green engineering [43].

## 8. Types of Niosomes

There are different types of niosomes, which are classified according to their size. Firstly, small unilamellar niosomes range from 10–100 nm. Another type of niosome is large unilamellar niosomes, ranging from 100–250 nm. On the other hand, there are multilamellar niosomes with a size range of 250–1000 nm. These different types of niosomes are obtained using different types of surfactants, charge inducers, stabilizers, and synthesis methods, with a direct effect on the nanoparticle size and morphology. Some examples of different types of niosomes are discussed below [1].

### 8.1. Proniosomes

Proniosomes are a type of niosome. These particles are the dehydrated, free-flowing form of niosomes. Proniosomes consist of a membrane-stabilizing surfactant and a coating carrier. These niosomes must be hydrated in an aqueous medium when in use, as the nonionic surfactant is miscible in water. This type of niosome has several advantages, preventing the aggregation and fusion of components and significantly reducing the problem of caking. It is extremely useful in transdermal administration of drugs, as it improves the flow capacity of the encapsulated drug, improving the administration accuracy [44].

### 8.2. Ethosomes

Surfactant ethosomes, like all niosomes, use a nonionic surfactant miscible with organic and inorganic polar solvents such as lower alcohols and water. This type of niosome also consists of phospholipids that become highly deformable when interacting with the solvent. Ethosomes are classified according to their composition, with classic ethosomes, binary ethosomes, and transethosomes. They are widely used in transdermal drug delivery because their components enhance efficient permeation by affecting the intercellular region [45].

### 8.3. Aspasomes

Aspasomes are lamellar vesicles to be incorporated into semi-solid preparations. They are generally composed of ascorbyl palmitate in water, to which diacetyl phosphate is added for molecule stabilization. When used, these niosomes require a moisturizing medium and sonication treatment. They have many applications in the pharmaceutical and cosmetic fields by improving transdermal permeation [46].

### 8.4. Discosomes

Discosomes, as their name suggests, are disc-shaped niosomes. This shape is the result of a niosome solubilization process, forming large ellipsoidal and disc-shaped vesicles. Their shape depends on the phase of the nonionic surfactant, forming discs of 11–60 μm. These discs are generally used as carriers for highly polar drugs, maintaining their stability in the face of heat fluctuations [47].

### 8.5. Polyhedral Niosomes

Polyhedral niosomes’ main feature is that they have a spherical shape but lack uniformity. This type of niosome is formed at low cholesterol concentrations, and membranes of these vesicles are generally gel-like, indicating that the hydrocarbon chains have minimal mobility, giving them an unusual angular shape if—and only if—they are above their phase transition temperature (43 °C), where their angular shape is not lost, and acquire a spherical shape, which, when cooled, results in morphological alteration due to thermal changes producing irreversible membrane changes [47].

### 8.6. PEGylated Niosomes

These are biodegradable, biocompatible, and non-immunogenic niosomes containing a polyethylene glycol modification, enabling them to escape capture by the mononuclear phagocytic system, resulting in longer circulation time for the administered drug and increasing its accumulation. They are generally used when the drug has poor solubility and low bioavailability [48].

## 9. Characterization of Niosomes

It is important to know the techniques that exist to characterize niosomes, since the information obtained will allow us to understand their properties and the potential they have as systems for the administration of bioactive compounds. Some parameters are size and shape through microscopy techniques, zeta potential, composition through FTIR, and stability, among others.

### 9.1. Size Analysis by Dynamic Light Scattering (DLS)

Dynamic light scattering (DLS), also known as photon correlation spectroscopy (PCS), is a widely used technique for characterizing the size of colloidal particles in suspension, such as niosomes. This methodology allows key physicochemical parameters to be determined, such as hydrodynamic radius, diffusion coefficient, polydispersity, and, in some cases, the spin radius. The operating principle is based on the interaction of a coherent light beam with particles in suspension, which are in constant Brownian motion. As the light is scattered, it is captured at a specific angle, and by analyzing the intensity fluctuations of the scattered light, the average size of the niosomes present in the system is calculated [49].

### 9.2. ζ Potential

The zeta potential (ζ) is an essential parameter in the characterization of colloidal stability of systems such as niosomes. It represents the difference in electrical potential between the particle surface and the diffuse layer of ions surrounding it, known as the electric dual layer. This potential, expressed in millivolts, indicates the degree of electrostatic repulsion between particles with similar surface charges. High values (positive or negative) suggest greater stability, as they decrease the probability of niosome aggregation or flocculation, which is crucial for maintaining their structural integrity and functionality in pharmaceutical or biomedical applications [50].

### 9.3. Polydispersity Index (PDI)

Polydispersity index (PDI) is a measure of heterogeneity in particle size within a sample of niosomes. This parameter is derived from DLS analysis and provides information on the size distribution present. A PDI close to 0 indicates a monodispersed sample, while higher values (>0.3) reflect greater variability in vesicle size. In physicochemical terms, polydispersity can also be interpreted as the ratio between average molecular weight by mass (Mw) and average molecular weight by number (Mn), indicating the structural uniformity of the system in niosomal formulations, a low PDI is desirable to ensure controlled and reproducible release of the active ingredient [51].

### 9.4. Fourier Transform Infrared Spectroscopy (FTIR)

Fourier transform infrared spectroscopy (FTIR) is a useful technique for structural characterization of niosomes, especially for confirming the presence of functional components such as surfactants, cholesterol, and encapsulated drugs. This technique operates in the infrared region of the electromagnetic spectrum, corresponding to wavelengths of 8 × 10^−5^ to 1 × 10^−2^ cm (energies of 4.6 to 46 kJ/mol). By applying the Fourier transform, a signal from the time domain is converted into a representation in the frequency domain, facilitating the identification of characteristic bands of molecular vibrations. Only vibrations that cause a change in the molecular dipole moment are active in IR, allowing the detection of specific functional groups present in the niosomal formulation [17].

### 9.5. Scanning Electron Microscopy (SEM)

**Figure 3:**
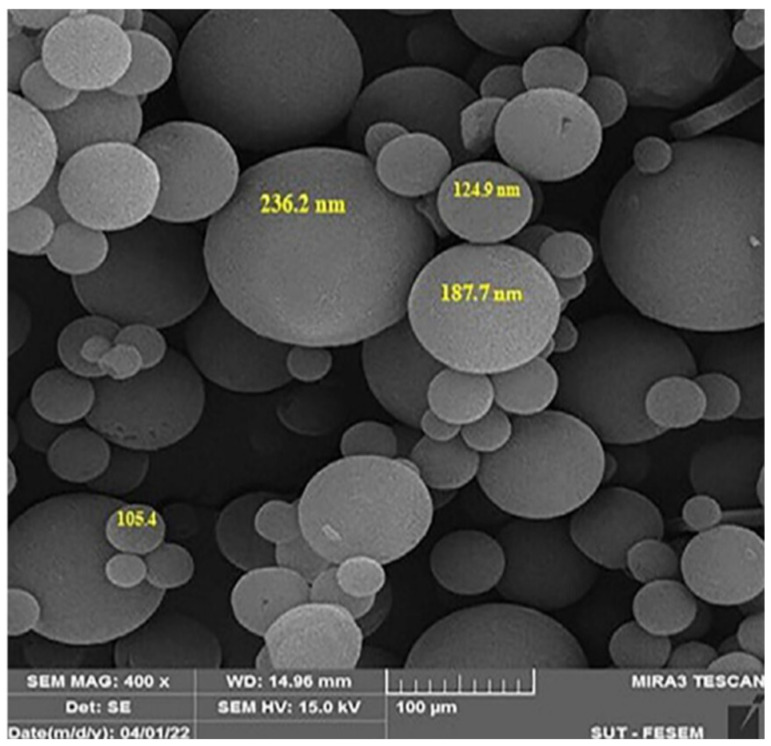
Morphological structure and size of niosomes by SEM (adapted from [53]).

Scanning electron microscopy (SEM) allows direct visualization of the surface morphology of niosomes. In this technique, an electron beam is directed toward the surface of the sample, generating different signals such as backscattered or secondary electrons, which are captured by specific detectors. These signals are amplified and processed to obtain high-resolution images. SEM provides detailed information on the shape, size, and surface distribution of niosomes, as shown in Figure 3, complementing the results obtained by techniques such as DLS. This morphological characterization is essential for validating the vesicle structural integrity and their potential applicability in controlled release drug systems [52,53].

### 9.6. Transmission Electron Microscope

**Figure 4:**
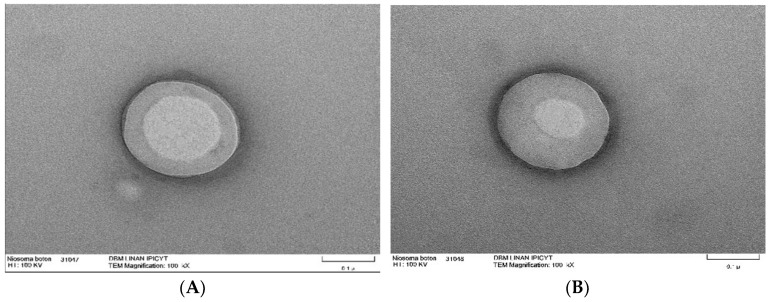
TEM micrographs of niosomes with encapsulated triacontanol. Niosomes with encapsulated triacontanol ((**A**,**B**) scale bar 0.1 μm).

Transmission electron microscopy is a tool used to examine the morphology of nanomaterials such as niosomes. With this type of microscope, obtaining images with specific intensities is possible due to the interaction of the electron beam with the sample. In this microscopy technique, a sample drop is deposited on copper grids coated with carbon, sometimes requiring staining, and is dried at room temperature. TEM allows for direct visualization of niosomes, identifying their shape. Additionally, it enables the observation of their internal structure, such as the organization of lipid layers. An example of this is niosomes synthesized from span60 and cholesterol, which have the ability to encapsulate triacontanol, a fatty alcohol with various biological activities (Figure 4) [54].

### 9.7. Confocal Laser Scanning Microscopy

**Figure 5:**
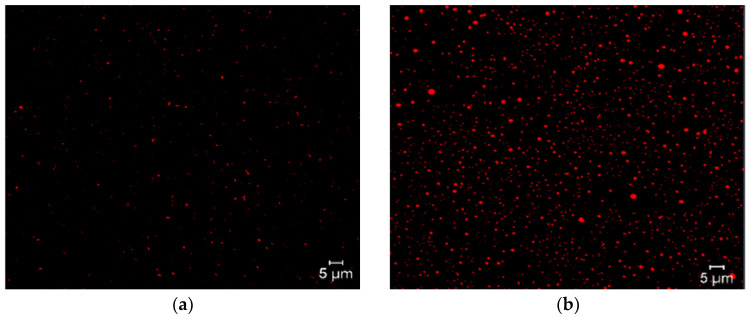
Micrographs of niosomes with Nile Red fluorochrome. Blank niosomes (**a**), niosomes with encapsulated molecules (**b**).

Confocal laser scanning microscopy is an innovative tool due to its image quality in macrometric niosome formulations, allowing visualization of the internal structure of lipid systems, as well as their structural integrity and lamellar organization. This tool can also obtain three-dimensional reconstructions of the vesicular system. Another advantage is the use of fluorochromes, which are molecules with the ability to selectively label different components of niosomes. These fluorochromes are inserted into the niosomes depending on their affinity and can be visualized under a microscope due to their fluorescent and solvatochromic nature. Examples of this include the interaction of surfactants with Nile Red, which is a hydrophobic fluorochrome, confirming that niosomes are formed and that the surfactant forms the lipid region, as shown in Figure 5 [54].

## 10. Applications of Niosomes

Niosomes have multiple therapeutic applications since they can be designed with special characteristics to transport compounds with biological activity such as antioxidants, anticancer agents, anti-inflammatories, and immunostimulants, among others (Table 1).

### 10.1. Delivery of Bioactive Compounds

One of the most widely used applications in nanotechnology is the encapsulation of bioactive compounds, as their stability, bioavailability, and biological activity are improved. Encapsulation of these compounds also improves release rates, maintains activity under extreme conditions, and reduces lipid oxidation. Encapsulation consists of enclosing bioactive substances within a carrier material. Bioactive compounds are naturally occurring compounds found in plants, with antioxidant, anti-inflammatory, and anticancer properties, among others. This activity is conferred by the molecular structure of their compounds. Thus, they are used to treat and prevent diseases. Examples of bioactive compounds that have been encapsulated include the following: antioxidants such as vitamins C and E; polyphenols such as flavonoids, quercetin, and phenolic acids; omega-3 and omega-6 fatty acids; carotenoids; and various benzoquinones such as perezone, which has demonstrated significant biological activity. Bioactive compounds can be unstable when exposed to light, temperature, and oxygen, and encapsulation is required to prevent degradation and increase biological activity [54].

### 10.2. Delivery of Anticancer Drugs

Cancer treatments present various difficulties such as untargeted administration, uncontrolled release, and drug-resistant tumor interactions. Niosomes have been used for the selective delivery of cytotoxic drugs to specific tumor sites with a high success rate. Encapsulated anticancer drugs improve their therapeutic effect due to altered pharmacokinetics and biodistribution while being more selective. These drugs can be delivered through different mechanisms depending on the type of tissue and the physicochemical characteristics of the niosome and the bioactive molecule. Passive delivery refers to the deposition of niosomes within the tumor due to cellular characteristics of the malignant neoplasm. On the other hand, active drug delivery refers to the structural modification of niosomes with a ligand or conjugate so that tumor cells capture the niosomes. Physical delivery is based on the conditions of the microenvironment where niosomes will be introduced, where the compound will be released through a physical stimulus. Due to the characteristics of the tumor environment, passive delivery facilitates the action of niosomes in these environments, as it facilitates the uptake of niosomes by cancer cells. For this purpose, adaptable molecules are used to synthesize functionalized niosomes that can specifically target tumor cells [55]. For example, the use of niosomes to encapsulate hydroxy-camptothecin and its intravenous administration in mice with S-180 tumors resulted in the complete elimination of tumors. Another example is the preparation of niosomes with paclitaxel and the preparation of PEG-gambogic acid niosomes for anticancer therapies and to improve the stability of gambogic acid. Niosomes have also been used in treatment strategies at different stages, such as in the encapsulation of hydrophobic curcumin and hydrophilic doxorubicin, which are released at different times, improving their cytotoxic effect through synergy against HeLa cell lines [56].

### 10.3. Immunologic Applications

Another innovative approach to niosomes has been the study of the immune response through the use of vaccines as a strategy to eliminate viruses and other pathogens. A new approach to vaccines is based on DNA and RNA antigens with the aim of overcoming the limitations associated with conventional vaccination strategies, such as low safety, the need for multiple doses, and side effects. However, due to antigen instability, the use of nanocarriers is required. The most commonly used nanocarriers are liposomes; however, liposomes are susceptible to oxidative degradation because they contain phospholipids in their structure. To overcome chemical stability problems, other options such as niosomes have been chosen. These carriers have been used for drug delivery and vaccines that improve the effectiveness of immunotherapy while reducing adverse effects. Niosomes have been used to encapsulate immunostimulatory drugs, which must be delivered appropriately to the target region. The use of niosomes for vaccine administration is a promising area due to the versatility of these nanocarriers in this field and the wide variety of surfactants that can be used for their formulation, in addition to preparation methods, obtaining niosomes with immunological selectivity [57].

### 10.4. Drug Carriers to the Central Nervous System

Neurodegenerative diseases are characterized by progressive loss of neuronal function, leading to motor impairment and cognitive decline. The efficacy of various therapeutic agents administered orally and parenterally is compromised by inefficient transport of drugs to the brain, mainly due to impediments such as the blood–brain barrier (BBB), which is a semipermeable membrane that prevents introducing dangerous components into the brain. The BBB also controls the uptake of nutrients and substances necessary for neuronal function. The transport of molecules across the BBB occurs through five different mechanisms: passive diffusion, paracellular transport, mediated transport by entry and exit proteins, receptor-mediated transcytosis, and adsorptive transcytosis. The BBB constitutes an obstacle to drug delivery, with only a few drugs able to cross it depending on their size, lipophilicity, and degree of dissociation. One solution is the use of lipid nanoparticles such as niosomes, which have proven capable of targeting the brain through ligand conjugation. Suitable ligands allow for active and targeted delivery of drugs to the central nervous system. These transport systems have a promising future in pharmaceutical applications, mainly through the nose–brain route [58,59].

Nonionic surfactant vesicles were introduced as an innovative and effective method for drug delivery. Their composition is mainly nonionic surfactants and cholesterol. They can be manufactured using different methods, which have a direct effect on their formation and physicochemical properties. The correct formulation of niosomes is important to achieve functionalization of the niosomal surface with affinity for specific proteins at target sites. Modification of their surface is relatively simple due to the functional groups that can be added to their hydrophilic heads. Due to their composition, niosomes exhibit biodegradability, which improves their biocompatibility by reducing the tendency to accumulate in organs.

Nanostructures can alter the characteristics and behavior of drugs after administration, which is generally carried out through passive diffusion, depending on lipophilicity and molecular weight. Nanostructures behave differently according to surface area, ligand binding, and the carrier. These features are essential for the treatment of diseases such as glioblastoma as well as other neurodegenerative disorders. Several studies have functionalized niosomes with certain ligands, such as glucose derivatives like N-palmitoylglucosamine, which have been developed as carriers for the delivery of the neuropeptide DynB and doxorubicin to the brain. In addition, niosomes containing folic acid and transferrin have been developed for delivery to the central nervous system [59,60]. Recent studies also address the use of niosomes for the treatment of gliomas, which are characterized by high incidence and mortality rates. Curcumin, a phenolic compound, has been encapsulated in niosomes to improve its efficacy as an anticancer agent, enabling it to cross the blood–brain barrier, prolong its release, and target tumor tissues. These niosomes have been functionalized with transferrin to achieve receptor-mediated targeting through the inhibition of P-glycoprotein by nonionic surfactants and active targeting toward glioma cells [61].

## 11. Conclusions

Niosomes are innovative nanocarriers composed of nonionic surfactants, cholesterol, charge inducers, and a hydration medium. These components provide physicochemical stability, improve compound solubility and permeation, and enhance cellular uptake by controlling drug release. Due to the versatility in modifying their physicochemical properties, niosomes are highly adaptable for various applications, ranging from cancer treatments and vaccines to cosmetic and ophthalmic products. The selection of surfactants, cholesterol concentration, pH, and preparation method plays a crucial role in obtaining functionalized niosomes with specific geometries and characteristics that address issues related to stability, bioavailability, and specificity. Understanding these structural features and preparation processes is key to designing efficient nanocarriers.

However, despite presenting lower toxicity compared to other nanocarriers, such as liposomes, niosomes may still pose risks, including immune responses and interactions with plasma proteins. These challenges highlight the need to design and synthesize biodegradable surfactants capable of providing the desired niosome geometries. Furthermore, functionalization with specific ligands is critical to achieve targeted delivery, alongside exploring alternative administration routes. Hybrid niosomes, composed of polymers or lipids, may offer a promising solution for achieving these goals.

One significant area of opportunity in niosome research lies in overcoming challenges related to the reproducibility and scalability of formulations. Variations in surfactant types, cholesterol concentration, and geometries across different production batches present an obstacle to achieving consistent and high-quality products. Addressing these variations through standardized production methods and enhancing the functionality of niosomes will be crucial for expanding their applications and improving their clinical and commercial potential.

## Figures and Tables

**Table 1:** Therapeutic applications of niosomes: advantages and disadvantages.

Application	Description	Advantages	Disadvantages
Delivery of bioactive compounds	Encapsulation of compounds such as antioxidants, anticancer agents, anti-inflammatories, and immunostimulants to improve their stability, bioavailability, and biological activity.	Improves the stability of compounds. Increase bioactivity and bioavailability. Protect against extreme conditions. Enhances controlled release of compounds.	Can require complex encapsulation processes. Long-term stability may be challenging.
Delivery of anticancer drugs	Niosomes for selective delivery of cytotoxic drugs to tumor sites. Uses passive, active, or physical mechanisms for controlled release.	Improves therapeutic efficacy. Increases drug selectivity. Can overcome drug resistance.	Limited availability of specific formulations. Challenges in modifying niosomes for precise delivery.
Immunological applications	Use of niosomes for encapsulation of vaccines and immunological drugs, improving immunotherapy effectiveness, and reducing side effects.	Potential to enhance immune response. Lower risk of side effects compared to liposomes. Versatility in formulation with different surfactants.	Antigen instability requires continuous encapsulation. May not be suitable for all vaccines or pathogens.
Drug delivery to the central nervous system	Niosomes to overcome the blood–brain barrier (BBB) and deliver drugs to the brain through ligand conjugation for active and targeted delivery.	Capable of crossing the blood–brain barrier (BBB). Enhances targeted delivery to specific areas of the brain. Potential in the treatment of neurodegenerative diseases	Transport across the BBB remains a challenge. Requires suitable ligands and complex formulations.

## Data Availability

All data are available in the manuscript.
